# Localization and interactions of *Plasmodium falciparum* SWIB/MDM2 homologues

**DOI:** 10.1186/s12936-015-1065-9

**Published:** 2016-01-20

**Authors:** Warren Antonio Vieira, Thérèsa L. Coetzer

**Affiliations:** Wits Research Institute for Malaria, Wits Medical School, 7 York Road Parktown, Johannesburg, 2193 South Africa; Plasmodium Molecular Research Unit, Department of Molecular Medicine and Haematology, School of Pathology, Faculty of Health Sciences, University of the Witwatersrand, Johannesburg, South Africa; National Health Laboratory Service, Johannesburg, South Africa

**Keywords:** SWIB/MDM2, *Plasmodium falciparum*, Heat stress response, Malaria, Programmed cell death, Apoptosis

## Abstract

**Background:**

Malaria remains a global health problem and the majority of deaths are caused by *Plasmodium falciparum* parasites. Due to the rapid emergence of drug-resistant strains, novel avenues of research on the biology of the parasite are needed. The massive proliferation of asexual, intra-erythrocytic parasites every 48 h could kill the human host prior to transmission of slow-developing gametocytes to the mosquito vector. A self-induced *P. falciparum* programmed cell death mechanism has been hypothesized to maintain this balance between the parasite and its two hosts, but molecular participants of the cell death pathway in *P. falciparum* have not been characterized. Proteins with SWIB/MDM2 domains play a key role in metazoan programmed cell death and this study provides the first evaluation of two parasite SWIB/MDM2 homologues, PF3D7_0518200 (*Pf*MDM2) and PF3D7_0611400 (*Pf*SWIB).

**Methods:**

The function of these proteins was assessed by predicting their structural topology with the aid of bioinformatics and determining their location within live transgenic parasites, expressing green fluorescent protein-tagged *Pf*MDM2 and *Pf*SWIB under normal and elevated temperatures, which mimic fever and which are known to induce a programmed cell death response. Additionally, *P. falciparum* phage display library technology was used to identify binding partners for the two parasite SWIB/MDM2 domains.

**Results:**

Structural features of the SWIB/MDM2 domains of *Pf*MDM2 and *Pf*SWIB, suggested that they are chromatin remodelling factors. The N-terminal signal sequence of *Pf*MDM2 directed the protein to the mitochondrion under both normal and heat stress conditions. *Plasmodium falciparum* phage display library technology revealed that the C-terminal SWIB/MDM2 domain of *Pf*MDM2 interacted with a conserved protein containing a LisH domain. *Pf*SWIB localized to the cytoplasm under normal growth conditions, while approximately 10 % of the heat-stressed trophozoite-stage parasites presented a rapid but short-lived nuclear localization pattern. Two *Pf*SWIB binding partners, a putative Aurora-related kinase and a member of the inner membrane complex, were identified.

**Conclusion:**

These novel data provide insight into the function of two parasite SWIB/MDM2 homologues and suggest that *Pf*MDM2 plays a role within the mitochondrion and that *Pf*SWIB is involved in a stage-specific, heat-stress, response pathway.

**Electronic supplementary material:**

The online version of this article (doi:10.1186/s12936-015-1065-9) contains supplementary material, which is available to authorized users.

## Background

The asexual intra-erythrocytic life cycle of *P. falciparum* lasts approximately 48 h, from invasion to egress, whereby a single merozoite can give rise to as many as 32 new merozoites, while the sexual gametocytes, which are transmitted to the *Anopheles* mosquito host, require up to 12 days to reach maturity [1]. In light of this, if all the newly formed merozoites were to invade erythrocytes every 48 h, the human host may die before gametocyte maturation has occurred. As the human immune system removes the parasite poorly there must be another means of regulation to protect the host from premature death. One such mechanism has been hypothesized as parasite self-induced, programmed cell death (PCD) [2].

Apoptotic features in the malaria parasite were first described in 1997 [3, 4], with the ‘crisis form’ of the parasite hypothesized as a PCD marker [4]. Subsequently, numerous studies have documented apoptosis and autophagy markers, including DNA laddering, loss of mitochondrial membrane potential, apoptotic body formation, and cytoplasmic vacuolization during various life stages of *Plasmodium* in response to a variety of stress stimuli [5]. Markers, such as DNA fragmentation and mitochondrial dysregulation, have been noted in cultured parasites under normal, non-limiting conditions, which suggests an intrinsic property [6]. To date, no experimentally proven PCD machinery has been described in the parasite, although several candidate genes have been identified by bioinformatics, including metacaspases [7] and SWIB/MDM2 domains [8].

The mammalian MDM2 protein, originally identified in transformed mice fibroblasts, contains several functional domains, including a SWIB/MDM2 domain [9, 10]. This anti-apoptotic protein is located primarily within the nucleus of unstressed cells where it binds to p53 via its N-terminal region, containing the SWIB/MDM2 domain. This interaction prevents p53 binding to DNA and induces the nuclear export, ubiquitination and proteasome-dependent degradation of p53 [11]. Under genotoxic conditions, numerous processes occur to stabilize p53, including MDM2 phosphorylation to prevent its association with p53, which brings about cell cycle arrest and, if required, cell death [11].

SWIB/MDM2 domains have also been identified in several other proteins and protein complexes, one such being the 2 MDa multi-subunit nuclear assembly, the SWI/SNF complex [12]. This ATP-dependent chromatin remodelling complex and transcriptional regulator, originally discovered in yeast, binds to DNA and hydrolyses ATP, which alters chromatin structure through nucleosome sliding and histone octomer insertion and/or ejection. The complex is composed of constant units, believed to be core functional units and includes the Swp73p/SNF12 protein containing a SWIB/MDM2 domain, as well as other apparently variable units, proposed to facilitate a degree of specificity and/or functionality [12]. The complex is involved in various stress response pathways, including exposure to elevated temperatures, heavy metals and metabolic inhibitors [13, 14]. In humans, a homologue of the SWI/SNF complex has also been shown to associate with p53 and regulate its activities, facilitating cell cycle halting and fine tuning the balance between repair and apoptosis induction [15–17]. The BAF60a protein of the complex is responsible for p53 binding, not through its C-terminal SWIB/MDM2 domain but rather directly via an N-terminal region [17].

SWIB/MDM2 domains participate in activities such as protein–protein [18] and chromatin-related interactions [19], but their precise functional role(s) in the cell are poorly characterized.

The *P. falciparum* genome encodes two putative SWIB/MDM2 domain-containing proteins: PF3D7_0518200, SWIB/MDM2 domain-containing protein, putative [PlasmoDB: PF3D7_0518200], which will be designated as *Pf*MDM2 in this study; and PF3D7_0611400, SWI/SNF-related matrix-associated actin-dependent regulator of chromatin [PlasmoDB: PF3D7_0611400], which will be designated as *Pf*SWIB.

This study aimed to assess the two *P. falciparum* SWIB/MDM2 homologues. Under normal and heat stress conditions *Pf*MDM2 localized to the mitochondrion, while the cytoplasmic *Pf*SWIB protein underwent a short-lived nuclear redistribution after heat stress. Biopanning revealed one or more binding partners for the SWIB/MDM2 domains of these two *P. falciparum* homologues, suggestive of transcriptional or stress pathway involvement.

## Methods

### Bioinformatics

Amino acid sequences of proteins containing SWIB/MDM2 domains were collected from a variety of prokaryotic and eukaryotic species—*Homo sapiens* [NCBI: ACX31156]; *Canis lupus familiaris* [NCBI: BAB11975]; *Mus musculus* [NCBI: AAB09030]; *Sus scrofa* [NCBI: ABV09038]; *Gallus gallus* [NCBI: AAF04192]; *Danio rerio* [NCBI: NP_571439.2]; *Pongo abelii* [NCBI: NP_001124685.1]; *Callorhinchus milii* [NCBI: AEW46991]; *Xenopus laevis* [NCBI: NP_001086070]; *Arabidopsis thaliana* [NCBI: AAP68331.1]; *Toxoplasma gondii* [NCBI: EEB00952.1]; *Taranis japonicus* [NCBI: ACS36127.1]; *Caenorhabditis elegans* [NCBI: CAA87424.1]; *Grosmannia clavigera* [NCBI: EFW99809.1]; *Drosophila melanogaster* [NCBI: AAF48235.1]; *Solanum chacoense* [NCBI: ABE11612.1]; *Homo sapiens* [NCBI: ACX31156.1]; *Zea mays* [NCBI: NP_001148297.1]. These were used for multiple EMBL-EBI clustal omega sequence alignments [20] against the two malaria proteins of interest. Multiple sequence alignments were expressed graphically using BioEdit Sequence Alignment Editor [21]. Percentage identity and similarity was calculated using EMBOSS Needle [22].

### Tertiary structure analysis

Structural modelling of the malaria proteins was conducted with the online servers of the Swiss Model Workspace Automatic Modelling Mode [23], PHYRE2 [24] and ESyPred3D Web Server 1.0 [25]. The following crystal and solution structures were used: the *Xenopus laevis* MDM2 SWIB/MDM2 domain [PDB id: 1YCQ chain A] [9], the *Mus musculus* SWI/SNF-related, matrix-associated, actin-dependent regulator of chromatin subfamily D member 1 SWIB/MDM2 domain [PDB id: 1UHR] [26], and the *Arabidopsis thaliana* SWIB/MDM2 domains [PDB id: 1V31 and 1V32] [27, 28]. Graphical display, orientation and colouring of various PDB files were conducted using the PyMOL Molecular Graphics System [29]. The quality of all the three-dimensional models was assessed by the QMEAN Server [30] and the best candidate models presented.

### Localization prediction

Various online prediction algorithms were employed to determine the likely cellular compartment to which the proteins would localize. These included cNLS Mapper [31]; MitoProt II—v1.101 [32]; NucPred [33]; PredSL; PSORT Prediction; PREDOTAR V1.03; PlasmoDB—PlasmoAP Results [34]; PATS Version 1.2.1 [35, 36]; PlasMit [37] and iPSORT Prediction.

### Parasite culture

The 3D7 strain of *P. falciparum* was cultured according to a slightly modified form of the standard protocol [38]. Briefly, parasites were maintained at 5 % haematocrit in culture medium (RPMI 1640 (Gibco BRL, Life Technologies Corp, CA, USA), supplemented with 0.5 % Albumax II (Life Technologies Corp), 0.21 % sodium bicarbonate, 50 mg/L gentamycin and 50 mg/L hypoxanthine). The culture medium was changed daily and optimal pH was maintained by daily gassing of the culture with 2 % O_2_, 5 % CO_2_ and 93 % N_2_. Sorbitol treatment was employed for asexual parasite synchronization [39].

Ethics clearance was obtained for this project. Ethics number M13-05-69; The University of the Witwatersrand; Committee for Research on Human Subjects (medical).

### PCR amplification and cloning

Genomic DNA was extracted from infected erythrocytes [40] and used for the amplification of various genes for both recombinant protein expression and transgenic parasite production. The primers, containing appropriate restriction endonuclease sites, are presented in the Additional files 1 and 2. PCR products and vectors were digested with the appropriate FastDigest^®^ restriction endonucleases (Thermo Fisher Scientific, Inc., Ueberlingen, Germany). The genes or gene domains were subsequently cloned into pARL2-GFP (donated by Dr Jude Przyborski, Marburg, Germany), pGEX-4T-2 or pET-15b vectors depending on the downstream application. After ligation, the pARL2-GFP constructs were used for the transformation of *Escherichia coli* XL10-Gold^®^ ultracompetent cells (Stratagene, CA, USA), while the Rosetta™ 2 (DE3) *Escherichia coli* line (Novagen Inc., WI, USA) was used for the pGEX-4T-2 and pET-15b constructs.

### Recombinant His- and GST-tagged protein expression and purification

Transformed colonies were grown in Overnight Express™ Instant TB Medium (Novagen Inc.), containing 100 μg/ml ampicillin and 50 μg/ml chloramphenicol, for 22 h at 250 rpm, at room temperature (~20 °C). The *Escherichia coli* cells were lysed by freeze-thawing and sonication and protein purification was conducted using the MagneGST™ and MagneHis™ kits (Promega, WI, USA), according to the manufacturer’s specifications. Depending on the downstream application, the purified fusion proteins could either be eluted from or retained on the magnetic beads. GST-tagged recombinant proteins were eluted in 150 μl of GST-Elution buffer (500 mM L-Glutathione, 500 mM NaCl, 50 mM Tris–HCl, pH 8.1) while the His-tagged recombinant proteins were eluted in 100 μl of His-Elution buffer (50 mM Na_2_HPO_4_/NaH_2_PO_4_ buffer, 150 mM NaCl, 0.5 M imidazole, pH 7.5 for *Pf*LisH and pH 7.8 for *Pf*ARK3). Protein concentration was determined using a bovine serum albumin (BSA) standard curve and molecular weight was assessed relative to a red cell membrane marker.

The eluted GST- and His-fusion proteins were dialysed at 4 °C against three changes of Tris buffered saline (TBS) (50 mM Tris–HCl, 150 mM NaCl, pH 7.5) or sodium phosphate buffer (50 mM Na_2_HPO_4_/NaH_2_PO_4_ buffer, 150 mM NaCl, pH 8), using a 10 kDa Slide-A-Lyzer MINI dialysis unit (Pierce Biotechnology Inc, IL, USA) for 30 min. The protein samples were then assessed by 12 % SDS–polyacrylamide gel electrophoresis (SDS-PAGE) [41] and immunoblotting, using a 1:25,000 Anti-GST horseradish peroxidase (HRP) conjugated primary antibody (Amersham Biosciences Ltd, Little Chalfont, UK) or 1:1200 Anti-His HRP conjugate primary antibody (Qiagen GmbH, Hilden, Germany).

### Biopanning against *Plasmodium falciparum* phage display libraries

*Plasmodium falciparum* phage display, mixed stage libraries were used according to a previous protocol, with slight modifications [42]. Briefly, a pre-screening step to eliminate background was carried out whereby the starting library (~1 × 10^7^ pfu/ml) was mixed with MagneGST beads containing at least 8 μg of recombinant GST, at room temperature for 1 h. The unbound phage were mixed with MagneGST beads bound to at least 8 μg of recombinant GST-*Pf*MDM2 or GST-*Pf*SWIB protein, for 1 h at room temperature. The beads were removed, washed five times with 2 ml of 0.05 % Tween-TBS for 10 min, and then added to 50 ml of log phase BLT5403 cells and incubated at 37 °C until lysis was noted. The lysate was then used as the starting library for the next round of biopanning. In total, four sequential rounds of biopanning were conducted to enrich for phage binding specifically to the recombinant *Pf*MDM2 and *Pf*SWIB proteins. After the final round, these phage were used for PCR analysis. The empty cassette PCR product was 216 bp and thus plaque PCR products ≥300 bp were sent for sequencing at Inqaba Biotec™, Johannesburg, South Africa.

### In vitro binding assays

In vitro binding assays were conducted to confirm the interactions identified using biopanning. One μg of the dialysed recombinant His-fusion binding partner protein, re-attached to 5 μl MagneHis beads, was exposed to increasing concentrations of the GST-tagged SWIB/MDM2 partner for 1 h at room temperature in a total volume of 150 μl TBS. The beads were washed three times in TBS and then the protein complexes were subjected to SDS-PAGE [40]. Control reactions were conducted as described above using equivalent amounts of recombinant GST protein as well as heat denatured (70 °C for 15 min) SWIB/MDM2 proteins to compensate for non-specific binding. The experiments were conducted in a reciprocal fashion, whereby dialysed recombinant GST-*Pf*MDM2 proteins were re-attached to MagneGST beads and exposed to increasing concentrations of the His-tagged binding partners. The resultant interactions were then assessed by 12 % SDS-PAGE and visualized by immunoblotting, as described above, or with Coomassie blue (0.05 % Coomassie Brilliant Blue R-250 (w/v), 25 % Isopropanol (v/v), 10 % acetic acid (v/v)). Protein concentrations were determined by densitometry, relative to a BSA standard, in the case of the latter technique.

### Transgenic parasite generation

Transfections were performed on synchronized ring-stage 3D7 *P. falciparum* parasites in a 2 mm BioRad Gene Pulser^®^ Cuvette (Bio-Rad Laboratories, CA, USA) with a minimum of 100 μg of plasmid DNA by electroporation using the Bio-Rad GenePulse Xcell™ electroporator (Bio-Rad Laboratories), at 310 V, with a resistance of 950 μF and a time of less than 15 ms. Positive WR99210 drug selection, at a final concentration of 2 nM, was applied and maintained after the first cycle of growth, as parasites which had taken up the pARL2-GFP construct would carry the human dihydrofolate reductase gene, providing resistance to the drug. The transgenic parasites were noted in cultures from 25 to 43 days after transfection.

### Heat shock analysis

Synchronized trophozoite stage transgenic parasites were exposed to 41 °C, a temperature equivalent to malaria-induced febrile illness, for 2 h and then returned to 37 °C. Previous work has indicated that such exposure induces a time-dependent apoptosis-like death mechanism [43]. The stressed parasites were viewed 30 min after the end of heat stress and then at set time points by fluorescence microscopy.

### Microscopy

For nuclear staining, parasites were treated with DAPI (Sigma-Aldrich Corp, MI, USA), at a final concentration of 0.2 μg/ml, or Hoechst 33258 pentahydrate (Invitrogen, CA, USA), at a final concentration of 6 μg/ml and incubated for 5 min at room temperature or 2 h at 37 °C, respectively, and then washed twice in culture medium.

To stain mitochondria, MitoSOX™ Red (Molecular Probes, Invitrogen, CA, USA) was used, which is selectively targeted to the mitochondria of living cells and then oxidized by mitochondrial superoxides [44] into a red fluorescent form. To verify that MitoSOX stains the mitochondria of *P. falciparum,* co-localization with MitoTracker Green FM (Molecular Probes, Invitrogen, CA, USA) was confirmed (see Additional file 3). This green fluorescent stain accumulates in the active mitochondria of *P. falciparum* [45], but it has almost identical emission and excitation properties as GFP, which prevented its use in this study. MitoSOX™ was added to 300 μl of cultured wild type 3D7 *P. falciparum* parasites at a final concentration of 0.5 μM and incubated for 15 min at 37 °C. Cells were washed twice with culture medium before visualization at 1000× magnification, using the BX41 Olympus Microscope system. The system included a U-MWU2 filter (excitation between 330 and 385 nm and emission above 410 nm, for DAPI visualization), a U-MWB2 filter (excitation between 460 and 490 nm and an emission above 510 nm, for GFP and MitoTracker Green FM visualization), U-MWG2 filter (excitation between 510 and 550 nm and emission maximum at 590 nm, for MitoSOX™ visualization), a U-25ND25 Olympus neutral density filter, an Olympus DP72 camera; and CellSense Dimensions 1.7 Software (Olympus Optical Corp Ltd, Tokyo, Japan). Images were combined and processed uniformly with Adobe Photoshop 7.0. The overlap coefficient (R) was determined for five parasites for each experimental procedure and averaged with the aid of Image J [46].

## Results

### *Plasmodium falciparum* SWIB/MDM2 domains conform to a helical cleft topology

Two broad groups of SWIB/MDM2 domains have been identified. The first group is involved in p53 binding and identified in MDM2 proteins, such as that found in the *Xenopus laevis* MDM2 protein [9]. The second group is identified in a variety of eukaryotic proteins and participates in chromatin remodelling, transcriptional regulation and unknown functions, such as that found in the *Saccharomyces cerevisiae* SNF12 protein [NCBI:CAA96302.1] and the *Arabidopsis thaliana* At1g31760 protein [NCBI:At1g31760] for example [19]. The *Plasmodium* Database [47] was screened using the BlastP search function for SWIB/MDM2 domain homologues. No matches were found relative to the domain identified in MDM2 proteins; however, low scoring matches were found to the SWIB/MDM2 domains document in non-MDM2 proteins. Two of these proteins, expressed by the parasite, are annotated as having SWIB/MDM2 domains—*Pf*SWIB and *Pf*MDM2—and these were selected for subsequent analysis.

**Fig. 1:**
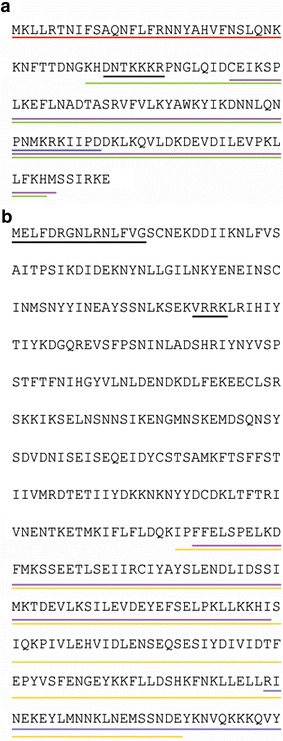
Signal sequence and domain predictions for *Pf*MDM2 and *Pf*SWIB. Signal sequences and functional domains within **a** full-length *Pf*MDM2 and **b** the N-terminus of *Pf*SWIB (first 420 amino acids). The *red line* indicates a putative mitochondrial localization signal sequence identified by the iPSORT Prediction algorithm with LLRTNIFSA being of critical importance; the *blue lines* indicate putative nuclear localization sequences identified by cNLS Mapper; the *black lines* indicate putative nuclear localization signal sequences identified by NucPred; the *purple lines* indicate the putative SWIB/MDM2 domains as defined on PlasmoDB. The *green line* represents the truncated form of the recombinant *Pf*MDM2 protein; while the *orange line* represents the recombinant region expressed for *Pf*SWIB

Predicted cellular localization signal sequences, as well as predicted functional domains within the two *P. falciparum* SWIB/MDM2 homologues are presented in Fig. 1. *Pf*MDM2 is annotated to contain a C-terminal SWIB/MDM2 domain, constituting most of the protein [48]. The same putative domain was annotated in the N-terminal region of *Pf*SWIB and was the only functional domain identified within this large protein of 830 amino acids [48]. Both domains were surrounded by a variety of monopartite and bipartite nuclear signal sequences predicted by different algorithms. A mitochondrial localization sequence was identified at the N-terminus of *Pf*MDM2.

**Fig. 2:**
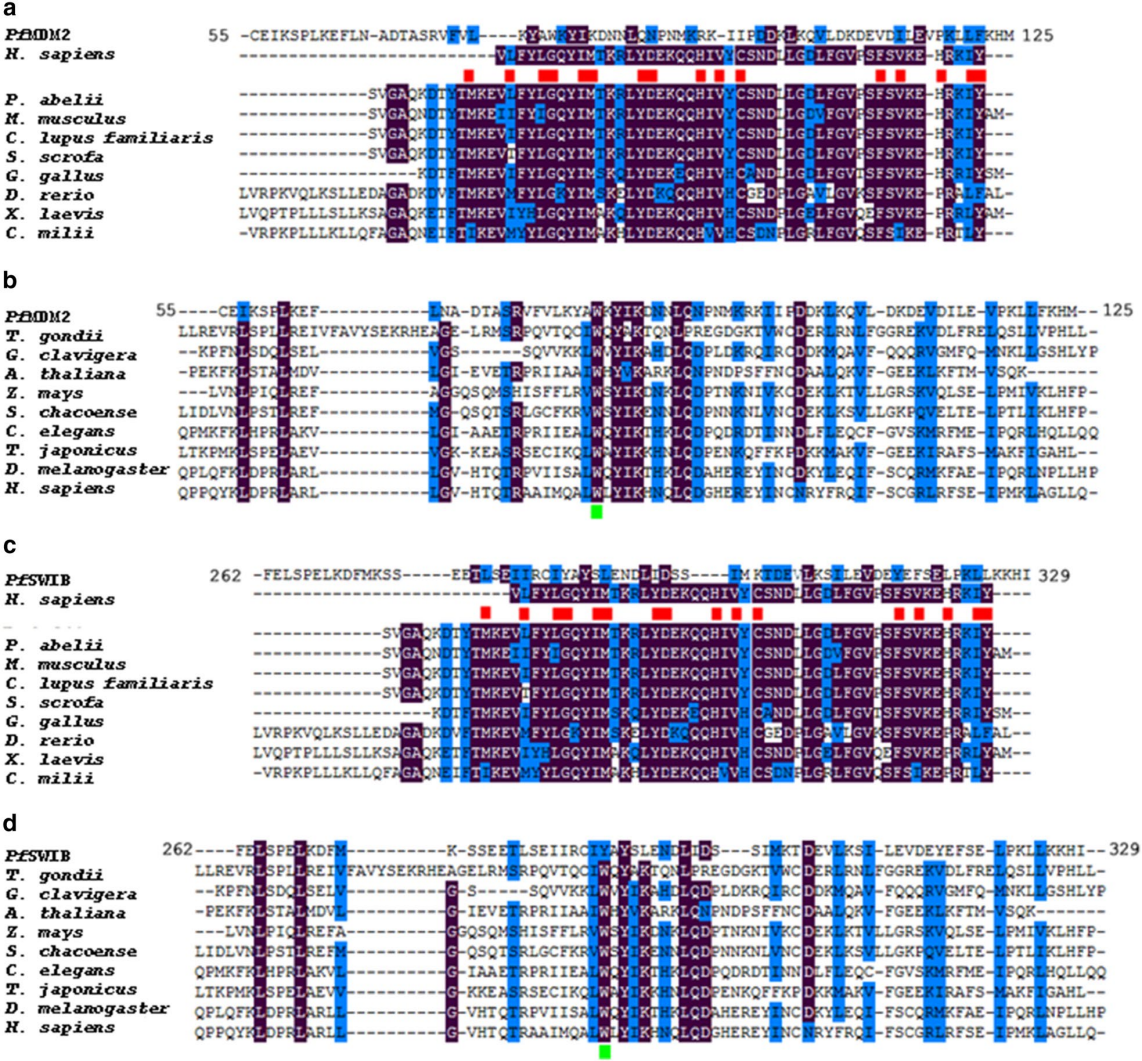
Clustal Omega primary amino acid sequence alignments for *Pf*MDM2 and *Pf*SWIB to various SWIB/MDM2 domains. Alignment of the *Pf*MDM2 SWIB/MDM2 to SWIB/MDM2 domains identified in **a** MDM2 proteins and **b** other proteins. Alignment of the *Pf*SWIB SWIB/MDM2 to SWIB/MDM2 domains identified in **c** MDM2 proteins and **d** other proteins. The *red blocks* indicate critical amino acid residues for p53 binding [9, 49], which show limited conservation in the two parasite domains. The *green blocks* highlight a previously described Trp residue conserved in SWIB/MDM2 domains, not found in MDM2 proteins [18]. *Purple blocks* indicate identical amino acids; *blue blocks* indicate similar amino acids; threshold set at 80 %

To further assess these candidates, primary sequence alignments were conducted using Clustal Omega while percentage identity and similarity was calculated using EMBOSS Needle. The SWIB/MDM2 domain has been retained during evolution [18]; therefore, to strengthen the analysis, proteins from several species ranging from *Homo sapiens* and other metazoans to unicellular organisms, such as *Toxoplasma gondii*, were used for this evaluation. The MDM2 domain of the *X. laevis* MDM2 protein shared 17.5 % identity and 35 % similarity and 19.2 % identity and 38.5 % similarity to the annotated domains of *Pf*MDM2 and *Pf*SWIB, respectively. The binding of MDM2 to p53 is mainly the result of van der Waals forces, facilitated by a high proportion of aromatic and hydrophobic residues within the SWIB/MDM2 domain [9, 49]—42.1 % in *Xenopus laevis* for example. The *P. falciparum* SWIB/MDM2 domains have a slightly lower hydrophobic and aromatic amino acid residue composition (39.4 % for *Pf*MDM2 and 40.6 % for *Pf*SWIB). Of the 16 residues marked as critical for p53 binding in the MDM2 domain (Fig. 2), only one is identical in each *P. falciparum* protein—Ile^83^ in *Pf*MDM2 and Asp^297^ in *Pf*SWIB—but there is a degree of semi-conservation. Furthermore there is a high degree of conservation of hydrophobic and/or aromatic residues (Fig. 2) that may facilitate a suitable environment for protein–protein interactions. The *P. falciparum* homologues could deviate in essential amino acids due to potential sequence and structural differences in their binding partner(s), relative to the metazoan p53 protein.

Similar to the *Xenopus laevis* protein, the MDM2 domain identified in the *Arabidopsis thaliana* SWI/SNF complex component SNF12 homolog [NCBI: Q9FMT4] shared 18.1 % identity and 36.1 % similarity and 24 % identity and 40.0 % similarity to the annotated domains of *Pf*MDM2 and *Pf*SWIB respectively. The residues of SWIB/MDM2 domains involved in chromatin remodelling and transcriptional processes are unknown. However, SWIB/MDM2 domains in MDM2 proteins have a conserved Gly residue whereas the domains in non-MDM2 proteins have retained a Trp residue in this position [18]. The Trp residue was conserved in *Pf*MDM2 (Trp^80^ marked in green in Fig. 2) but not *Pf*SWIB.

Overall sequence similarity was greater than identity in all these alignments, which correlates to a greater likelihood of homology as certain residue exchanges may have little or no effect on tertiary structure and/or protein function [50]. Protein structure, as opposed to sequence, often shows greater conservation during evolution [50, 51] and was, therefore, characterized for both *P. falciparum* SWIB/MDM2 homologues.

**Fig. 3:**
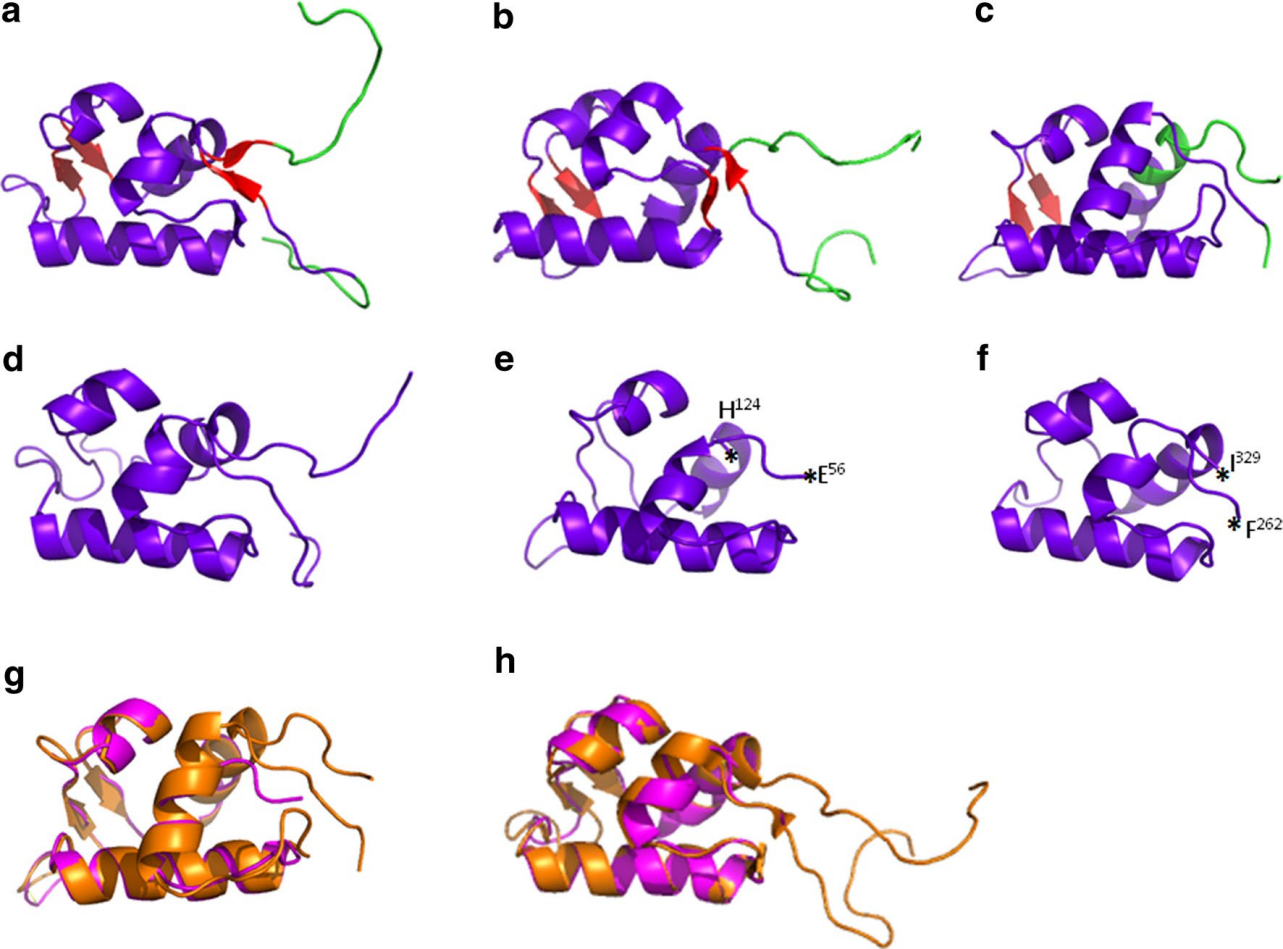
Molecular modelling of *Pf*MDM2 and *Pf*SWIB. Purple and red regions denote amino acids comprising the SWIB/MDM2 domain while *green regions* denote flanking regions. *Purple regions* represent alpha helices and random coils while *red regions* specifically highlight the beta sheets of the structure. **a** Solution structure of the SWIB/MDM2 domain of *Mus musculus* SWI/SNF related, matrix-associated, actin-dependent regulator of chromatin subfamily D member 1 protein (1UHR); **b** Solution structure of the *Arabidopsis thaliana* SWIB/MDM2 domain of the hypothetical protein At5g14170 (1V31); **c** Crystal structure of the *Xenopus laevis* SWIB/MDM2 domain of the E3 ubiquitin-protein ligase MDM2 protein (1YCQ, chain **a**); **d** Putative SWIB/MDM2 domain of yeast SNF12 modelled against 1UHR using the PHYRE2 modelling algorithm; **e** Putative SWIB/MDM2 domain of *Pf*MDM2 modelled against 1YCQ using the EsyPred modelling algorithm, Qmean score 0.835, 13.7 % identity, annotated residues denote the start and end of the modelled domain, which are indicated by *stars*; **f** Putative SWIB/MDM2 domain of *Pf*SWIB modelled simultaneously against 1UHR, 1V31 and 1V32 (solution structure of the SWIB/MDM2 domain of the hypothetical protein At5g08430 from *Arabidopsis thaliana)* using the PHYRE2 modelling algorithm, Qmean score 0.745, 28, 24 and 28 % identity to each template respectively, annotated residues denote the start and end of the modelled domain, which are indicated by *stars*. **g** Overlay of the modelled *Pf*MDM2 domain (*magenta*) against 1YCQ (*orange*); **h** Overlay of the modelled *Pf*SWIB domain (*magenta*) against 1V31 (*orange*)

Neither *P. falciparum* SWIB/MDM2 domains has been crystallized. Predicted tertiary structures were generated using several online tools—the Swiss Model Workspace Automatic Modelling Mode [23], PHYRE2 [24] and ESyPred3D Web Server 1.0 [25]. Predictions were made relative to the server’s inherent template databases or to user-defined templates. All algorithms predicted a helical cleft topology, comparable to that of crystallized and soluble structures of SWIB/MDM2 domains, for both malaria proteins. The most reliable model for each, based on Q-mean analysis whereby a score closer to 1 represents a more reliable model, showed that *Pf*MDM2 (Fig. 3e) and *Pf*SWIB (Fig. 3f) had good Q-mean values and were similar to crystallized or soluble structures of SWIB/MDM2 domains (Figs. 3a–c). They aligned well when overlaid (Fig. 3g–h). However, both of these predicted structures lacked the presence of beta-sheets, involved in capping the classical SWIB/MDM2 ‘twisted cleft’ topology [9]; although, the SWIB/MDM2 domain of the yeast Swp73p/SNF12 protein [NCBI: P53628] also lacked beta sheets when modelled by the PHYRE2 algorithm (Fig. 3d). The modelled domain of *Pf*MDM2 was composed of residues 56–124, which was two amino acids smaller than the annotated SWIB/MDM2 domain (residue 55–125).

### *Pf*MDM2 localized to the mitochondria under normal and heat stress conditions

The episomal pARL2-GFP expression system utilizes the *crt* promoter, allowing for continuous high level *Pf*MDM2-GFP expression during all stages of the asexual intra-erythrocytic cycle, to aid in localization studies. However, focus was placed on the late asexual stages, since the transcriptional profile of *Pf*MDM2 showed the highest mRNA expression in late trophozoites and schizonts, and current proteomic data has documented the presence of the protein in schizonts and salivary gland sporozoites [52, 53].

**Fig. 4:**
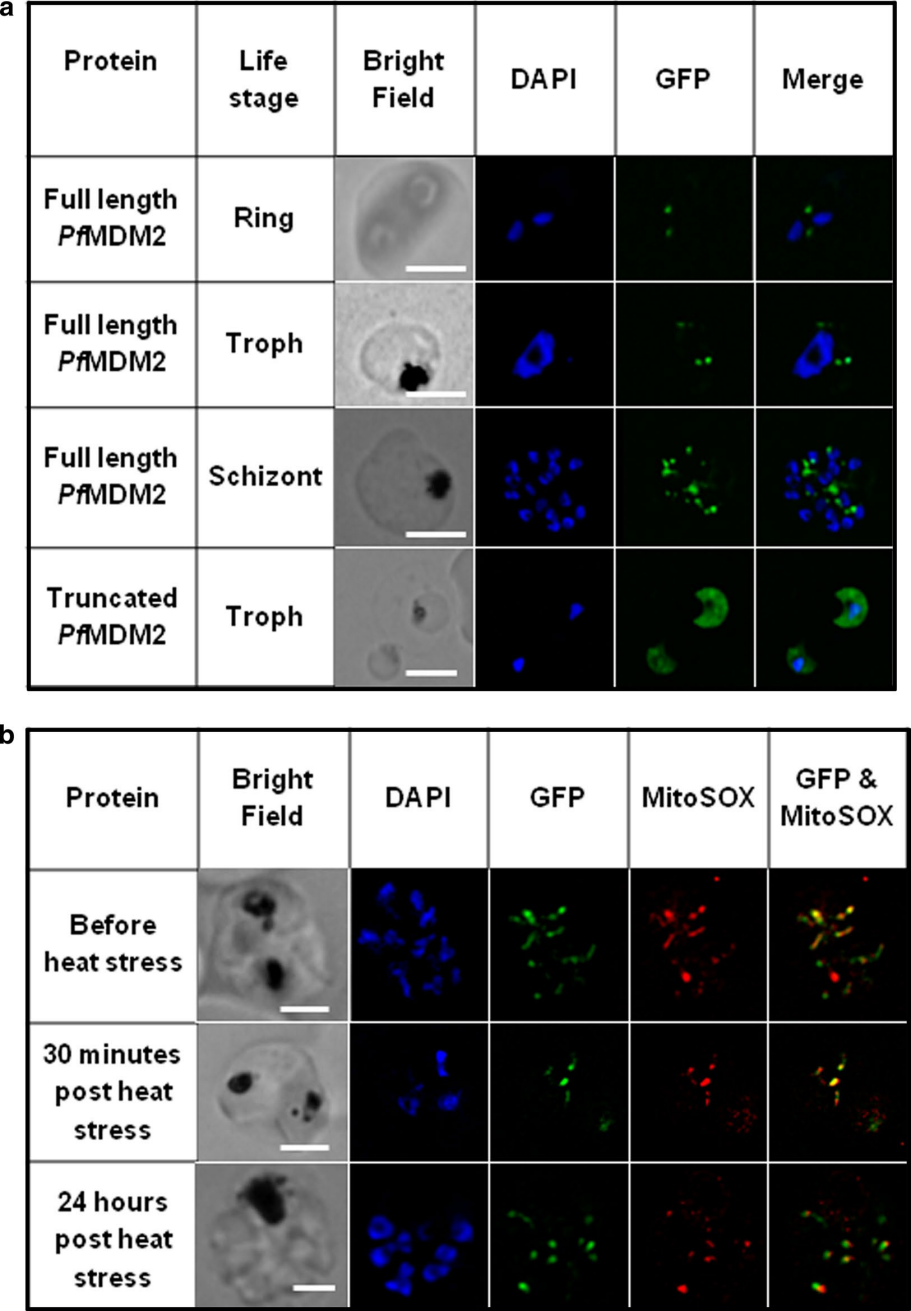
Mitochondrial localization of GFP-tagged *Pf*MDM2 within live *Plasmodium falciparum* parasites, directed by the protein’s N-terminus. **a** Live imaging of the *Pf*MDM2-GFP transgenic *P. falciparum* parasites under normal conditions. A distinct dot-like GFP signal was observed for *Pf*MDM2, adjacent to the *blue* DAPI-stained nucleus, increasing in number with the progression of the life cycle. The truncated *Pf*MDM2 protein lacking the N-terminus showed cytoplasmic distribution of the GFP signal. **b** Live co-localization of GFP-tagged *Pf*MDM2 protein to the mitochondria of late schizont stage *P. falciparum* parasites under normal conditions and 30 min and 24 h after the termination of heat stress (41 °C for 2 h). *R* ring stage, *T* trophozoite stage, *S* schizont stage. *Scale bar* in bright field represents 2.5 μm

As presented in Fig. 4, a discrete, non-nuclear, localization pattern was observed in all the intra-erythrocytic asexual life stages. The number of GFP focal points increased with an increase in the nuclei of late stage schizonts (Fig. 4a) and MitoSOX confirmed mitochondrial localization. The correlation coefficient always greater than 0.9 (Fig. 4b; correlation coefficient (R) 0.94). The truncated *Pf*MDM2-GFP protein lacking the N-terminal section and the predicted mitochondrial localization signal sequence (Fig. 1) remained in the cytoplasm (Fig. 4a). Although the protein was predicted to contain several strong nuclear localization sequences, the experimental data revealed that these sequences were inactive under the described conditions.

Heat-stress has previously been shown to induce PCD markers in *P. falciparum* [43]. The full-length *Pf*MDM2-GFP protein was retained (correlation coefficient always greater than 0.9) within the mitochondria of late stage parasites, 30 min after the termination of heat stress at 41 °C for 2 h (Fig. 4b; correlation coefficient (R) 0.93). After 24 h (Fig. 4b) the red MitoSOX™ staining pattern was similar to that of *Pf*MDM2-GFP but when overlaid the signals appeared to have shifted slightly; however, analysis with Image J still showed that co-localization was retained (correlation coefficient (R) 0.92). This shift may have been the result of the rapid cellular movement of the parasite or could indicate that the protein had moved out of the mitochondrion. The surviving parasites were, as expected, delayed in their development and were still in the late trophozoite stage.

### *Pf*SWIB shuttled between the cytoplasm and the nucleus under heat stress

**Fig. 5:**
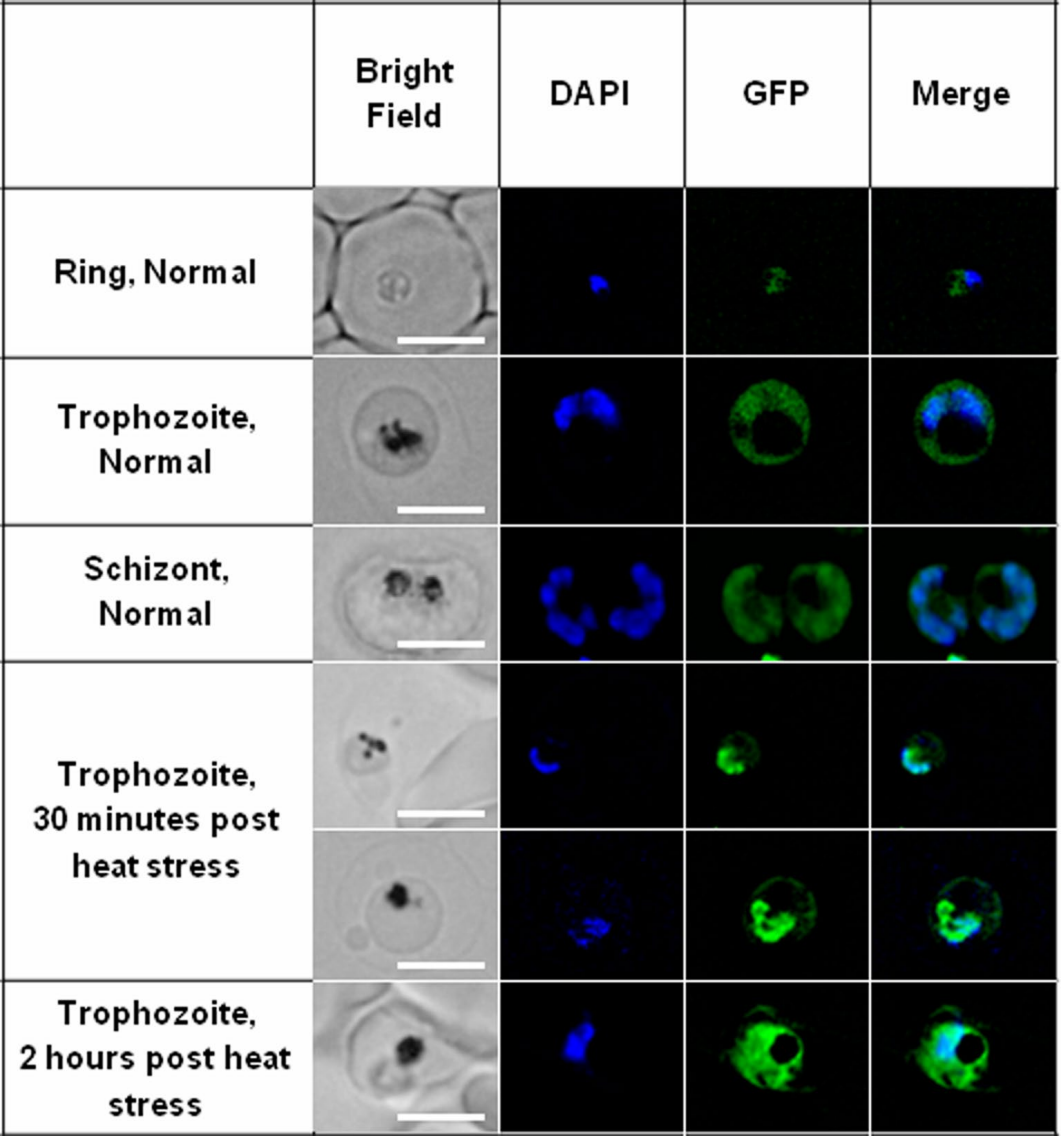
Live cellular localization of GFP-tagged *Pf*SWIB within *Plasmodium falciparum*. A distinct cytoplasmic GFP signal was observed in all life stages under normal conditions,whereas exposure to 41 °C for 2 h resulted in nuclear localization, 30 min after termination of heat stress. This redistribution was short lived since a nuclear signal was absent 2–24 h afterwards. *R* ring stage, *T* trophozoite stage, *S* schizont stage. *Scale bar* in bright field represents 2.5 μm

Transcriptome analysis revealed that *Pf*SWIB mRNA was expressed throughout the intra-erythrocytic life stages. Current proteomic data indicate that the protein was only detected in the late erythrocytic life stages, gametocytes and salivary gland sporozoites; however, it could still be expressed throughout the life cycle as the data are not complete [52, 53]. The use of the episomal pARL2-GFP expression system utilizing the *crt* promoter, would allow for continuous high level *Pf*MDM2-GFP expression during all stages of the asexual intra-erythrocytic cycle, to aid in this localization study. Focus was, therefore, again placed on late asexual intra-erythrocytic parasites, which showed cytoplasmic localization of *Pf*SWIB-GFP under normal growth conditions (Fig. 5). When PCD was induced by exposure to 41 °C for 2 h, there was a redistribution of *Pf*SWIB-GFP to the nucleus, 30 min after termination of heat stress (correlation coefficient (R) always greater than 0.9). This nuclear GFP signal was only noted in roughly 10 % of the stressed trophozoites and is likely due to the activation of one of the protein’s predicted nuclear localization signals. From 2 h (Fig. 5) onwards after the termination of heat stress no nuclear signal was evident within the population. Nucleolar and/or chromatin localization has been documented for SWIB/MDM2 homologues in response to stress [11, 13, 54], although, in this study such discrete localization could not be determined.

### Interactions of the SWIB/MDM2 domains of *Pf*MDM2 and *Pf*SWIB

**Fig. 6:**
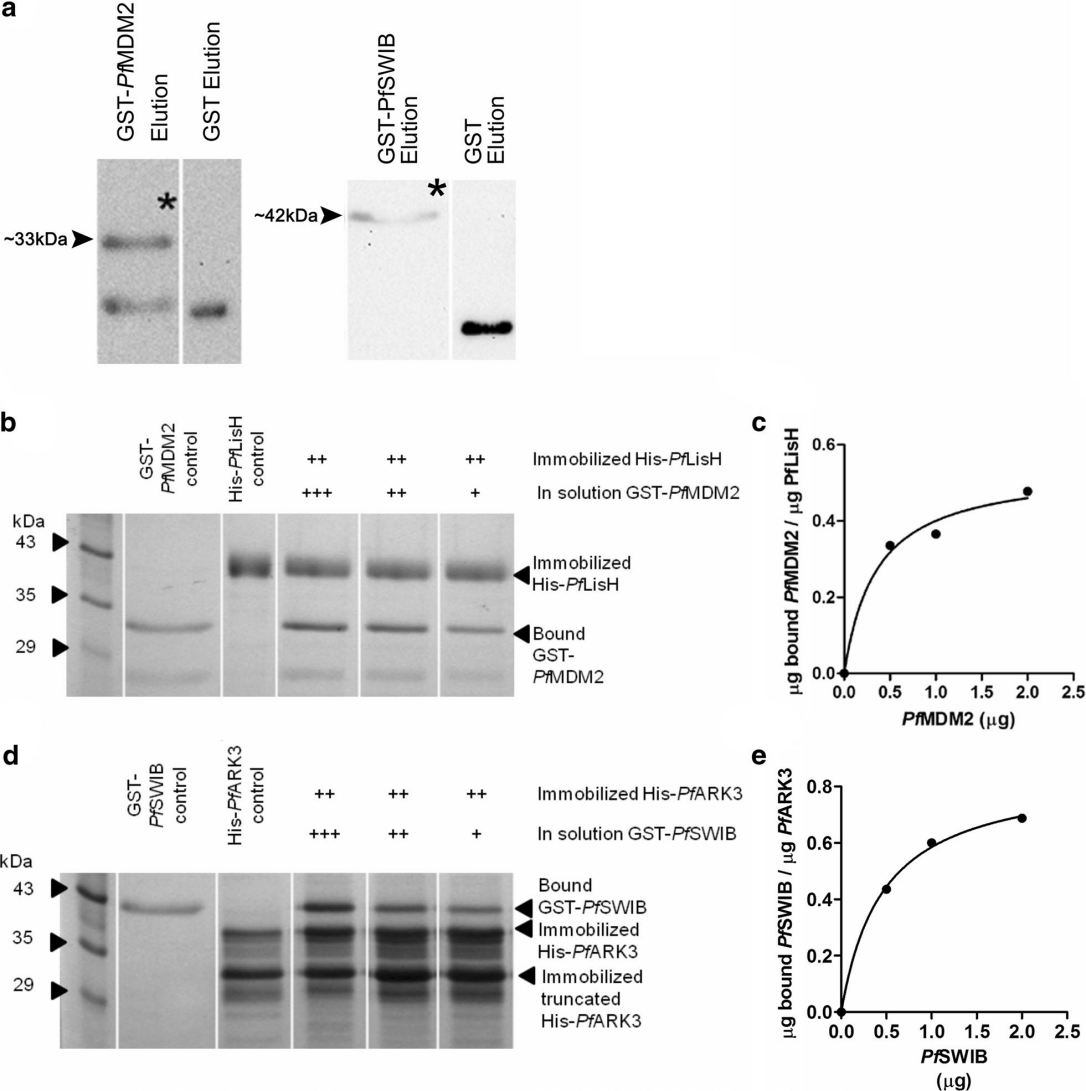
In vitro binding assays showed a concentration dependent interaction between *Pf*LisH and *Pf*MDM2. Representative images of **a** isolated and purified recombinant GST-*Pf*MDM2 and GST-*Pf*SWIB, migrating at ~33 and ~42 kDa, respectively, and identified by Western blot analysis using an anti-GST antibody (marked with *star*); **b** stained SDS polyacrylamide gels and **c** binding curves showing the concentration-dependent association between a constant amount of immobilized *Pf*LisH and increasing amounts of *Pf*MDM2; and **d** stained SDS polyacrylamide gels and **e** binding curves showing the concentration-dependent association between a constant amount of immobilized *Pf*ARK3 and increasing amounts of *Pf*SWIB

The SWIB/MDM2 domains of *Pf*MDM2 and *Pf*SWIB were expressed and purified as recombinant GST-fusion proteins. SDS-PAGE and immunoblotting revealed that they migrated at ~33 and ~42 kDa, roughly their correct molecular masses of 36 and 44 kDa (Fig. 6a), respectively. *Pf*MDM2 expressed as a full-length protein, but a small amount of a truncated form of ~24.5 kDa, not much bigger than the 23 kDa GST tag alone, was also produced.

The two SWIB/MDM2 recombinant domains were used for binding partner identification by phage display library technology (Table 1)—of the binding partners identified, three were assessed further. A central region of the PF3D7_1303400 protein [PlasmoDB: PF3D7_1303400] bound to *Pf*MDM2. This conserved 1022 amino acid protein of unknown function contains an N-terminal Lissencephaly type-1-like homology (LisH) domain [48], situated 466 amino acids upstream from the *Pf*MDM2 binding site and this protein will be designated as *Pf*LisH. Bioinformatic predictions revealed a strong possibility of nuclear localization for this protein.

**Table 1:** Binding partners for *Pf*MDM2 and *Pf*SWIB identified by biopanning of *Plasmodium falciparum* phage display libraries

Protein	Identity of the binding partner (total number of amino acids)	Amino acid sequence of binding domain (location in protein)	Annotated domains within binding partner (location in protein)	Region of the binding partner cloned into a His-tag vector
*Pf*MDM2	PF3D7_1303400—conserved *Plasmodium* protein, unknown function (1022)	KKKKKKEQTNEG KKSVKGINKKDK KRNSKVESKK (505–538)	LisH domain (8–34)	485–664
*Pf*SWIB	PF3D7_1356800—serine/threonine protein kinase, putative (ARK3) (4044)	IYEKVNIDNDKV KKKNLHSINDKK IKINKTFMNEKD MKGNNRKKYNTE KRDNIKRNENDN EKK (788–847)	Ser/Thr protein kinase domain, putative (1282–1528)	601–922
*Pf*SWIB	PF3D7_1003600—membrane skeletal protein IMC1-related (ALV5) (281)	PKTIIQEKIIHV PKNVTHIVEK IVEVPEVKYIEK IVEVPHIHYKNK YVPK (58–107)	Inner membrane complex protein domain (58–153)	2–241

Two binding partners were identified for *Pf*SWIB. Firstly, an N-terminal region of PF3D7_1356800 [PlasmoDB: PF3D7_1356800], a putative serine/threonine protein kinase (Aurora-related kinase 3 (*Pf*ARK3)) [48] was identified and the binding domain was 434 amino acids upstream of the putative kinase domain. Bioinformatic analysis as part of this study suggested nuclear localization for this protein. Secondly, an N-terminal region, correlating to part of the inner membrane complex domain of PF3D7_1003600 [PlasmoDB: PF3D7_1003600], a membrane skeletal protein (Alveolin 5 (*Pf*ALV5)) [48] was identified, which localizes to the inner membrane complex of the parasite [55].

The biopanning-identified binding domains of these three proteins were directionally cloned into the pET-15 vector (Table 1). Only His-*Pf*LisH and *Pf*ARK3 expressed as soluble recombinant proteins in sufficient quantity to verify the biopanning interactions. The His-*Pf*LisH protein was identified by immunoblotting using an antibody directed against the His-tag and it migrated at ~41 kDa, roughly double its expected molecular mass of 23.5 kDa (Fig. 6b, lane 3). The construct contained the correct sequence, so the aberration was probably due to the two low complexity regions in the recombinant protein, which affected the binding of SDS [56]. The His-*Pf*ARK3 protein (Fig. 6d, lane 3), migrated at its appropriate molecular weight of 40 kDa and a prominent truncated form of ~31 kDa, still containing the N-terminal His tag, was also expressed. Based on the difference in molecular weight, the truncated protein would likely have lost about 75 C-terminal amino acids, including only about three amino acids from the binding domain identified by biopanning. In vitro binding assays were conducted with the GST-tagged protein in solution and the immobilized His-tagged protein, and also in a reciprocal manner. Two independent binding assays for each protein set confirmed a concentration-dependent association between *Pf*MDM2 and *Pf*LisH, as well as *Pf*SWIB and *Pf*ARK3 (Fig. 6). Heat denatured ligand and GST protein alone were included as negative controls to ensure the specificity of binding. The controls revealed <25 % non-specific binding.

## Discussion

SWIB/MDM2 proteins have been localized to the mitochondria, nucleus, chloroplast, and cytoplasm within a variety of organisms and their functional pro-survival and stress response roles in the nucleus are well documented [11, 13, 14, 17, 18, 19, 54]. However, nothing is known about the functions of the *P. falciparum* protein homologues. To investigate this, several molecular and bioinformatic analyses were performed, including the localization of GFP-tagged *Pf*MDM2 and *Pf*SWIB in transgenic parasites under both normal and heat stress conditions, and biopanning to identify binding partners.

### The predicted tertiary structures of *Pf*MDM2 and *Pf*SWIB show similarities to SWIB/MDM2 domains

Bioinformatic analysis of SWIB/MDM2 domains identified in a variety of organisms has led to the idea that all extant forms share a common evolutionary ancestor with similar protein–protein interactions [18]. Crystallized SWIB/MDM2 domains exhibit ‘twisted cleft’ topologies constituted by four helices, creating a ‘barrel’, capped by one or more beta sheets [9, 26, 27, 28]. The SWIB/MDM2 domain is lined with numerous hydrophobic and aromatic residues (46.8 % of the amino acids for human MDM2), to create a suitable environment for the interaction with the transactivation domain of p53, mainly through van der Waals forces [9]. Two of the helices and one of the beta sheets of the ‘twisted cleft’ are involved directly in p53 binding [9].

The *P. falciparum* SWIB/MDM2 domains have similar, although slightly lower, hydrophobic and aromatic amino acid composition (39.4 % for *Pf*MDM2 and 40.6 % for *Pf*SWIB) and were predicted to form the ‘barrel’ of the classical SWIB/MDM2 structure but lacked beta-sheets. Modelling of the yeast SWIB/MDM2 domain, involved in chromatin remodelling [57], gave a similar result, suggesting that the ‘capping’ portion of the structure is not common to all SWIB/MDM2 domains. These data suggest that the parasite proteins are SWIB/MDM2 homologues.

### The role of *Pf* MDM2 in the mitochondrion

This study revealed mitochondrial localization of *Pf*MDM2 under normal and heat stress conditions (Fig. 3), indicating that *Pf*MDM2 is not implicated in a nuclear heat-induced PCD response. However, *Pf*MDM2 localization could not be assessed in those parasites which had died and a role of the protein in PCD of that sub-population could not be evaluated. In *Arabidopsis*, two out of six group I SWIB proteins, At1g31760 [NCBI: ABD59092.1] and At2g35605 [NCBI: AEC09127.1], containing only a SWIB/MDM2 domain, have been localized to the mitochondria, but their functions are unknown [19]. The *P. falciparum* genome shares similarities with *Arabidopsis thaliana* [58] and EMBOSS Needle alignment of the SWIB/MDM2 domain of *Pf*MDM2 revealed strong conservation of residues in At1g31760 (39 % identity and 51.9 % similarity) and At2g35605 (43.3 % identity and 55.8 % similarity). Thus, it can be speculated that *Pf*MDM2 plays a similar role to At1g31760 or At2g35605 within the mitochondrion. *Pf*MDM2 could be involved in transcriptional regulation, as part of a larger complex, since the mitochondrial genome codes for three genes [44].

A single phage display interaction (*Pf*LisH) was documented for *Pf*MDM2, which could indicate that this domain is limited in its interactions or it could reflect the fact that the phage display libraries used are not representative of the entire transcriptional profile of the parasite throughout its intra-erythrocytic life cycle [59]. In addition, immobilization of the *P.falciparum* SWIB/MDM2 domain on magnetic beads may limit its interaction with the phage. Finally, the SWIB/MDM2 domain on its own may not be sufficient for interactions that require the entire protein or specific post-translational modifications [59].

The *Pf*LisH mRNA and protein expression profile coincides with that of *Pf*MDM2 [52, 53], but the cellular location of *Pf*LisH is unknown, although bioinformatics strongly suggests nuclear localization, which makes an in vivo association under heat stress unlikely. *Pf*MDM2 has several predicted nuclear localization signals, possibly allowing it to traffic to the nucleus under other stressful conditions, providing an opportunity to interact with *Pf*LisH.

LisH motifs have been identified in 114 eukaryotic proteins from yeast to humans [60], and this commonly N-terminal domain participates in a variety of cellular processes, including transcriptional regulation, although it does not possess any known DNA binding motifs [61]. Of interest are the human TBL1 and the yeast Sif2p proteins. TBL1 interacts with the N-terminal SANT (SWI3/ADA2/N-CoR/TFHIIB) domains of the nuclear receptor co-repressor protein which is present in a variety of chromatin-associated complexes, including the SWI/SNF complex [62, 63].

If *Pf*MDM2 does move to the nucleus under specific conditions it could be hypothesized that *Pf*LisH and *Pf*MDM2 interact as part of a larger gene transcription complex. In the case of yeast Sif2p, its deletion increases mortality in response to starvation, especially under heat stress, which supports a role in gene-regulated stress resistance [64]. Therefore, a potential interaction of *Pf*MDM2 with *Pf*LisH in the nucleus may reflect a response to a physiological stress other than heat.

### To live or let die: that is the question for *Pf*SWIB

This study highlighted the unexpected localization of *Pf*SWIB to the cytoplasm under normal growth conditions; the rapid but short-lived nuclear localization in response to elevated temperatures (Fig. 5), and a novel binding partner (Fig. 6; Table 1). These data suggest three possible scenarios pertaining to the function of *Pf*SWIB.

### Could *Pf*SWIB be involved in parasite survival?

Previous work has documented that only a small number of late-stage parasites, undergoing heat stress, survive in vitro 24 h later [43], and this may correlate to the small number of trophozoite parasites showing a nuclear *Pf*SWIB-GFP signal. Elevated temperatures may activate one or more of the predicted nuclear localization signals, possibly by phosphorylation which is known to regulate nuclear import in other proteins [65]. The binding partner, *Pf*ARK3, is a putative atypical aurora-related serine/threonine protein kinase [66] and has a mRNA and protein expression pattern correlating to the late intra-erythrocytic asexual life stages—coinciding with the expression of *Pf*SWIB [52, 53]. *Pf*ARK3 lacks any annotated signal sequences but bioinformatic analysis in this study suggests nuclear localization, although such predictions should be treated with caution.

The human Aurora kinase A aids in inactivating and promoting the degradation of p53 through phosphorylation [67, 68], and a putative p53 homologue has been identified in the parasite genome [8]. It could thus be speculated that *Pf*SWIB and *Pf*ARK3 participate in preventing p53-mediated apoptosis in the nucleus in response to heat stress. Alternatively, *Pf*SWIB may influence parasite survival through transcriptional regulation by directing the phosphorylation activities of *Pf*ARK3, as human Aurora kinase B and its yeast homologue play key roles in gene transcription through histone H3 phosphorylation [69, 70]. However, although there was a considerable drop in the parasitaemia 24 h after heat stress, more than 10 % of the population still remained, suggesting that this cellular phenomenon could be pro-survival response in a parasite sub-population.

### Could *Pf*SWIB be involved in parasite death?

The SWIB/MDM2 homologue BAF60a of the mammalian SWI/SNF complex induces p53-directed apoptosis [17] and the parasite *Pf*SWIB protein may play a similar stage specific pro-PCD role, whereby transient nuclear localization triggers removal of those parasites. Several studies have shown that elevated temperatures >38.5 °C significantly inhibited the development and growth of intra-erythrocytic asexual parasites, which showed several features of necrosis and/or PCD [5]. This event is hypothesized to reduce the host’s parasite burden to prevent premature host death before effective parasite transmission [2, 43, 71]. Furthermore, interactions with *Pf*ARK3 may influence parasite survival through transcriptional regulation by possible histone phosphorylation [69, 70]. Fever is linked to schizont rupture and merozoite re-invasion, with the early life stages surviving in vitro heat stress better than late stages [5, 43, 71, 72]. Thus, removal of any lagging trophozoite stage parasites, each capable of giving rise to as many as 32 new parasites, could have a significant impact in decreasing the number of parasites. The schizonts, which did not show nuclear *Pf*SWIB-GFP localization, may have passed the point of susceptibility to *Pf*SWIB-directed PCD.

### Could *Pf*SWIB play a non-PCD role?

Although the protein conforms to a similar three-dimensional topology as other SWIB/MDM2 homologues, it does not imply a direct PCD role. A group I SWIB protein, At3g48600 [NCBI: AEE78436.1], has been documented in the cytoplasm of *Arabidopsisthaliana* [19]. EMBOSS Needle alignment of this protein with the SWIB/MDM2 domain of *Pf*SWIB revealed 22.5 % identity and 42.7 % similarity, suggesting that these two proteins may share similar, as yet unknown, functions in the cytoplasm.

One of the roles of *Pf*SWIB may be related to heat stress regulation. Firstly, the absence of Swp73p/SNF12 in yeast resulted in temperature-sensitive mutants, highlighting the involvement of this SWIB homologue in heat stress transcriptional regulation [57]. Secondly, in other organisms, similar migration patterns for heat stress participants in response to the addition or removal of heat stress, has been documented [73]. Thirdly, association between *Pf*AVL5, a member of the inner membrane complex [55], and *Pf*SWIB may assist in compartmentalizing the *Pf*SWIB protein until stress induces nuclear localization, another feature of heat stress participants [74–76]. A discrete localization pattern to the inner membrane complex was not documented for *Pf*SWIB, which may be a consequence of *Pf*SWIB-GFP protein over-expression, using the *crt*-promoter driven expression system. Fourthly, the SWI/SNF complex is involved in transcriptional initiation of heat shock protein 70 genes in humans and yeast [77, 78] and *Pf*SWIB may facilitate such transcriptional regulation [69, 70].

## Conclusion

The concept of PCD in *P. falciparum* is supported by the presence of biochemical markers in parasites subjected to stressful conditions. However, there is currently no knowledge of the genes and pathways involved in this process. This study provides novel insight by evaluating the molecular functions of two SWIB/MDM2 homologues, *Pf*MDM2 and *Pf*SWIB, which are potential PCD participants. It provides the first description of their localization within the parasite and their response to elevated temperatures, which induce PCD markers in parasites and which mimic fever periods experienced by malaria patients. Novel interactions with other parasite proteins were also identified. Bioinformatics suggested that the proteins were chromatin remodelling family members, deviating slightly from the typical twisted cleft topology of this group but structurally similar to the yeast SWIB/MDM2 homologue. Unexpectedly, *Pf*MDM2 showed N-terminal-directed mitochondrial localization under both normal and heat-induced PCD conditions. *Pf*SWIB localized to the cytoplasm under normal conditions, but after heat stress, it revealed a short-lived nuclear localization in a sub-population of trophozoites. Interestingly, some SWIB homologues in *Arabidopsis thaliana* also localize to either the mitochondria or the cytoplasm, which suggests that *Pf*MDM2 and *Pf*SWIB may play similar roles to these proteins, as opposed to metazoan SWIB/MDM2 homologues. Based on the data from this study, it is hypothesized that *Pf*MDM2 is involved in mitochondrial maintenance and gene expression, possibly as part of a larger transcriptional complex. Furthermore, it is postulated that *Pf*SWIB may have a stage-specific, pro-survival function and participate in the heat stress response of the parasite.

## Additional files

10.1186/s12936-015-1065-9 PCR primers for the amplification of *Plasmodium falciparum* genes/domains.
10.1186/s12936-015-1065-9 PCR primers for the amplification of binding partners for directional cloning into the pET-15b vector.
10.1186/s12936-015-1065-9 Live imaging of *Plasmodium falciparum* schizonts with Mitotracker™ FM Green and MitoSOX™ showing mitochondrial co-localization (Correlation coefficient (R) 0.91). Scale bar in bright field represents 5 μm.

## Abbreviations

PCD: programmed cell death
MDM2: mouse double minute 2
SWI/SNF: switch/sucrose non fermentable
SWIB: SWI/SNF complex B
p53: protein 53
BAF60a: BRG1-associated factor 60a
LisH: lissencephaly type-1-like homology (LisH)
ARK: aurora related kinase
AVL: alveolin
HRP: horse radish peroxidase
BSA: bovine serum albumin
