# Supplementary material for: Localization and interactions of Plasmodium falciparum SWIB/MDM2 homologues
Source: Malar J. 2016 Jan 20;15:32. doi: 10.1186/s12936-015-1065-9 (PMC4721021; doi:10.1186/s12936-015-1065-9)
Supplement: Supplementary file 1 — 10.1186/s12936-015-1065-9 PCR primers for the amplification of Plasmodium falciparum genes/domains. [file 12936_2015_1065_MOESM1_ESM.docx]

**Additional file 1** PCR primers for the amplification of *P. falciparum* genes/domains

| Gene | Domain | Vector | Primer Direction | Primer sequence (5’ to 3’)† | Size of PCR product (bp) |
| --- | --- | --- | --- | --- | --- |
| *Pf*MDM2 | putative MDM2/SWIB domain | pARL2-GFP | Forward | TCA CTC GAG ATG GGA AAA CAT GAT AAT ACG AA | 303 |
|  |  |  | Reverse | AGG CCT AGG ATG TTT AAA TAA CAA TTT TGG AA |  |
| *Pf*MDM2 | putative MDM2/SWIB domain | pGEX-4T-2 | Forward | TCA GGA TCC ATG AAT ACG AAA AAA AAA AGA CCA A | 303 |
|  |  |  | Reverse | TCA CTC GAG TCA TGA CAT ATG TTT AAA TAA CAA |  |
| *Pf*MDM2 | entire gene | pARL2-GFP | Forward | CCT CTC GAG ATG AAA CTT TTG AGA ACA AAC A | 414 |
|  |  |  | Reverse | ACT CCT AGG TTC CTT TCG AAT AGA TGA CAT A |  |
| *Pf*SWIB | putative MDM2/SWIB domain | pGEX-4T-2 | Forward | CCC GGA TCC ATC CCT TTT TTT GAA CTA TCT | 474 |
|  |  |  | Reverse | ATT CTC GAG TCA TTC ATC ATT GGA ACT CAT TTC ATT |  |
| *Pf*SWIB | entire gene | pARL2-GFP | Forward | GGA CTC GAG ATG GAA CTA TTT GAT AGA GGA AA | 2508 |
|  |  |  | Reverse | GCG CCT AGG AAA ATT ATT ATT ATT ATT ATT ATT ATT ATT ATT GTT |  |

† Restriction site in forward primers (underlined): For pARL2-GFP: *Xho*I cleavage site (CTC GAG), and for pGEX-4T-2: *BamH*I (GGA TCC). Restriction site in the reverse primers (underlined): For pARL2-GFP: *Avr*II cleavage site (CCT AGG); and for pGEX-4T-2: *Xho*I cleavage site (CTC GAG).

The restriction sites are preceded by several random nucleotides to enhance the efficiency of digestion by the restriction endonucleases.
