# Supplementary material for: Localization and interactions of Plasmodium falciparum SWIB/MDM2 homologues
Source: Malar J. 2016 Jan 20;15:32. doi: 10.1186/s12936-015-1065-9 (PMC4721021; doi:10.1186/s12936-015-1065-9)
Supplement: Supplementary file 2 — 10.1186/s12936-015-1065-9 PCR primers for the amplification of binding partners for directional cloning into the pET-15b vector. [file 12936_2015_1065_MOESM2_ESM.docx]

**Additional file 2** PCR primers for the amplification of binding partners for directional cloning into the pET-15b vector.

| Gene | Primer Direction† | Primer sequence (5’ to 3’) | Size of PCR product (bp) |
| --- | --- | --- | --- |
| *Pf*LishH | Forward | TCT GTC CAA GAT CAT ATG AGT AAT TGT AGT AGT ACA ACC T | 573 |
|  | Reverse | GCC GGA TCC TCA TAT GGG TGC TTT AAT TTG TT |  |
| *Pf*ARK3 | Forward | ACA GAA ACT CAT ATG AAA ACT TTA CAA GAA GAG GTA AAT GAA | 1020 |
|  | Reverse | AGA GGA TCC TTA TGA CTT AGC TGA TGA TGA TAA TAA GA |  |

† Restriction site in forward primers (underlined) for pET-15b: *Nde*I cleavage site (CAT ATG). Restriction site in the reverse primers (underlined) for pET-15b: *BamH*I cleavage site (GGA TCC).

The restriction sites are preceded by several random nucleotides to enhance the efficiency of digestion by the restriction endonucleases.
