# Supplementary figures and images for: Localization and interactions of Plasmodium falciparum SWIB/MDM2 homologues
Source: Malar J. 2016 Jan 20;15:32. doi: 10.1186/s12936-015-1065-9 (PMC4721021; doi:10.1186/s12936-015-1065-9)

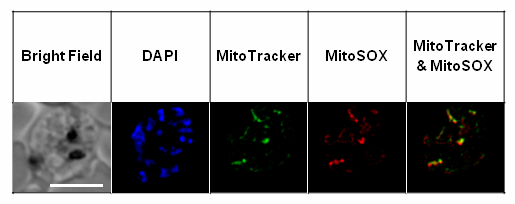

Supplement: Supplementary file 3 — 10.1186/s12936-015-1065-9 Live imaging of Plasmodium falciparum schizonts with Mitotracker™ FM Green and MitoSOX™ showing mitochondrial co-localization (Correlation coefficient (R) 0.91). Scale bar in bright field represents 5 μm. [file 12936_2015_1065_MOESM3_ESM.png]
